# Novel Insight into Dugong Mortality: First Report of Systemic *Achromobacter xylosoxidans* Infection, Disseminated Intravascular Coagulation, and Associated Pathogenesis

**DOI:** 10.3390/ani15162441

**Published:** 2025-08-20

**Authors:** Piyaporn Eiamcharoen, Piyarat Khumraksa, Santi Ninwat, Tatsawan Suttiboon, Narissara Keawchana, Peerapon Sornying, Watcharapol Suyapoh

**Affiliations:** 1 Faculty of Veterinary Medicine, Kasetsart University, Bangkok 10900, Thailand; piyaporn.e@ku.th; 2 Marine and Coastal Resources Research Center (Lower Andaman Sea), Trang 92150, Thailand; piyarat.lmbc@gmail.com (P.K.); niwat@hotmail.com (S.N.); 3 Marine and Coastal Resources Research Center (Lower Gulf of Thailand), Songkhla 90100, Thailand; tatsawanunderthesea@gmail.com; 4 Faculty of Veterinary Science, Prince of Songkla University, Songkhla 90110, Thailand; narissara.k@psu.ac.th (N.K.); peerapon.s@psu.ac.th (P.S.); 5 Dugong and Seagrass Research Station, Prince of Songkla University, Songkhla 90112, Thailand

**Keywords:** *Dugong dugon*, *Achromobacter xylosoxidans*, pathology, oxidative damage, disseminated intravascular coagulation, captive rehabilitation

## Abstract

Dugongs (*Dugong dugon*), classified as vulnerable marine mammals, are increasingly impacted by infectious diseases, yet the role of septicemia and disseminated intravascular coagulation (DIC) in their mortality remains uncharacterized. This study aimed to investigate the pathological and microbiological features associated with an acute mortality event in a juvenile dugong during rehabilitation in southern Thailand. Comprehensive histopathological and microbiological analyses were conducted on tissue samples collected postmortem. Bacterial isolation and identification were performed using standard culture techniques and the VITEK-2 system. Histological examination revealed multisystemic lesions, including fibrin thrombi, hemorrhage, hepatocellular degeneration, pancreatic necrosis, lymphoid depletion, and neuronal damage. Oxidative stress and DNA damage were confirmed in brain tissues through immunofluorescence detection of 4-hydroxynonenal (4-HNE) and 8-hydroxy-2′-deoxyguanosine (8-oxodG). *Achromobacter xylosoxidans*, an opportunistic pathogen, was isolated from multiple organs, consistent with acute systemic infection. These findings represent the first evidence of septicemia-associated DIC in dugongs caused by *A. xylosoxidans*, highlighting a previously undocumented cause of mortality in dugongs. The results emphasize the role of opportunistic bacteria in triggering oxidative damage and coagulopathy and underscore the importance of early detection and targeted therapeutic strategies to improve survival in stranded or rehabilitated dugongs.

## Simple Summary

Dugongs are endangered marine mammals found in coastal areas of South-east Asia and Australia. In this study, we report the sudden death of a dugong calf that was rescued and cared for at a rehabilitation center in Thailand. Despite receiving treatment for dehydration and an eye condition, the calf died after developing signs of a severe and rapidly progressing illness. A detailed examination revealed a serious infection caused by *Achromobacter xylosoxidans*, a bacterium commonly found in water. The infection had spread throughout the body. We also found evidence of brain injury caused by harmful molecules known as reactive oxygen species. The bacteria were resistant to several antibiotics, which emphasizes the need for greater awareness in future rehabilitation practices. This is the first reported case of such an infection in a dugong. The findings raise concern about secondary opportunistic infections by this environmental bacterium, which may contaminate medical instruments, water systems, or even be transmitted through human contact. Understanding these risks can help improve infection prevention and the survival chances of stranded marine mammals in care.

## 1. Introduction

Dugongs (*Dugong dugon*) are the sole extant species of the Dugongidae family within the order Sirenia. According to the International Union for Conservation of Nature (IUCN) Red List of Threatened Species, global dugong populations are considered vulnerable to extinction [1]. This species is primarily found in a limited range extending from East Africa and the Middle East, across Southeast Asia to New Caledonia, and latitudinally from Okinawa Island in Japan to Australia [2,3]. Thailand is a renowned habitat for endangered dugongs, particularly around Muk and Talibong Islands in Trang Province, located in the Andaman Sea, where notable populations are found [4]. Currently, dugongs face various anthropogenic threats, including targeted or incidental captures, vessel collisions, and habitat degradation [5], in addition to non-anthropogenic challenges, such as infectious diseases [6], as similarly observed in cetaceans [7,8,9,10].

Infectious diseases are major contributors to the mortality of cetaceans and sirenians across various regions, including the Canary Islands, the Andaman Sea of Thailand, the Philippines, Brazil, Italy, and the Netherlands [6,7,8,11,12,13]. Many of the bacteria involved are pathogenic, and a considerable number are opportunistic organisms naturally harbored by marine mammal hosts [14,15]. Several of these bacteria, such as *Leptospira* spp., *Mycobacterium* spp., and *Vibrio* spp. have the potential to cause pathological changes and may also act as zoonotic agents, posing risks to both animal and human health [16,17,18]. Necropsy examinations of stranded cetaceans and sirenians often reveal infectious and inflammatory conditions, frequently associated with septicemia [6]. Instances of septicemia have been linked to several opportunistic bacterial pathogens, including *Streptococcus dysgalactiae*, *Morganella morganii*, and *Erysipelothrix rhusiopathiae* [19,20]. These pathogens typically arise in compromised individuals, often as a result of underlying factors such as parasitic infestations or environmental stressors [21]. Coagulation disorders are a recurrent and potentially fatal complication of septicemia, which can progress to disseminated intravascular coagulation (DIC) [22]. It has been reported primarily in pinniped species, such as New Zealand sea lions (*Phocarctos hookeri*) [23] and Northern elephant seals (*Mirounga angustirostris*) [24]. However, septicemia-induced DIC in dugongs during rehabilitation and the associated etiology of sepsis remain unreported.

This study aims to characterize the clinical, pathological, and microbial findings in a live stranded dugong calf with systemic *A. xylosoxidans* infection, to determine the pattern of oxidative damage and DIC associated with the infection.

## 2. Materials and Methods

### 2.1. Animal Signalment and History

On 10 August 2024, a live stranded male dugong calf was discovered on the east side of Koh Poda, Krabi Province, Thailand. Immediate assistance was provided, and the calf was transported by boat from the stranding site to Nopparat Thara Pier, then by truck to the nursery station at the Natural Resources and Environment Institute, Rajamangala University of Technology Srivijaya, in Trang Province. The dugong calf had a body length of 102 cm and weighed 13.8 kg. Based on the body length and weight assessment, the calf was classified as a neonate, which typically exhibit body length ranging from 0.97 to 1.24 m and weight between 14 and 34 kg, according to Marsh et al. (1980) [25]. The observed dugong calf displayed small and short vibrissae sparsely distributed around the perioral region. Minor callosities were noted along the edges of the pectoral flippers. The umbilicus was completely closed. No dark green staining was observed on the horny plates of the upper and lower jaws within the oral cavity. Additionally, no fetal folds were present along the ventral surface. Based on these characteristics, the calf was estimated to be approximately two months old [25]. Upon initial examination, the calf was weak, dehydrated, and in poor bodily condition, with an estimated body condition score of 2/5, which was assessed according to the Dugong Necropsy Manual in Thailand [26]. In brief, the BCS assessment involved evaluating three parameters—cervical depression, dorsal musculature, and lateral body profile—and classifying the animal into one of five categories. A BCS of 1/5 (very thin) is characterized by a deep neck groove, a sharp vertebral outline, and an emaciated body profile; a BCS of 2/5 (thin) presents with a visible neck groove, a flattened back, and reduced body fullness; a BCS of 3/5 (healthy) shows no obvious neck groove, a slightly flattened but full back, and a gently rounded body profile; a BCS of 4/5 (overweight) is defined by the absence of a neck groove, a fully rounded back, and increased girth; and finally, a BCS of 5/5 (obese) is indicated by the absence of a neck groove, a broad and rounded back, and a markedly distended body. Clinical signs, including a concave neck, persistent suckling behavior indicative of hunger, and lethargy, suggested that the calf had been separated from its mother for several days. While precise estimation of the duration of fasting in dugong calves is limited due to the lack of species-specific data, these findings are consistent with at least 2–3 days of inadequate milk intake. Such signs reflect progressive undernutrition and dehydration, as documented in other mammalian and marine mammal neonates [27,28]. The left eye was opaqued, and the calf was unable to maintain balance in an upright position. The initial treatment focused on ocular therapy; however, the dugong calf developed acute clinical deterioration after one week of rehabilitation. Systemic enrofloxacin was administered empirically at a dosage of 5 mg/kg, intramuscularly, once daily (SID) from 21 August 2024 until death, based on clinical judgment and prior experience, as antimicrobial susceptibility results were not available during treatment. Despite 17 days of extensive therapy and rehabilitation, the animal’s condition continued to decline, and the calf ultimately died. A necropsy was performed for further investigation, and antimicrobial susceptibility testing was conducted postmortem.

### 2.2. Necropsy

The dugong calf was necropsied following a standardized procedure [29,30]. Tissue samples were collected from multiple organs and anatomical sites, including the entire brain, trachea, lungs, heart, liver, gallbladder, pancreas, spleen, kidneys, adrenal glands, urinary bladder, lymph nodes, thymus, stomach, tongue, esophagus, intestines, skin from the area anterior and superior to the right eye, skeletal muscle with traumatic lesions, and both eyes, to enable comprehensive pathological evaluation. All specimens were subsequently preserved in 10% neutral buffered formalin [31].

### 2.3. Bacteriological Examination

Bacteriological analysis was performed on tissue samples from the lung, liver, spleen, kidney, brain and heart. The tissues were cultured on MacConkey agar (Oxoid, Thermo Fisher Scientific, Waltham, MA, USA) and incubated at 35 °C in a 5% CO_2_ atmosphere. Additionally, the samples were inoculated directly onto blood agar (Oxoid) and incubated at 35 °C. Further bacterial identification and characterization were conducted using the VITEK-2 Compact system (bioMérieux, Marcy l’Etoile, France) [32]. No polymerase chain reaction (PCR) assays were performed for bacterial identification. Antibiotic susceptibility testing was performed using the Kirby–Bauer disk diffusion method. Bacterial cultures were grown overnight on Mueller–Hinton (MH2) agar plates. Colonies were suspended in nutrient broth, and the turbidity of the suspension was measured using an optical spectrophotometer (λ = 600 nm). The optical density (OD) was adjusted to 0.25 or 0.50. A sterile cotton swab was dipped into the bacterial suspension, and a bacterial lawn was applied to the surface of the agar plates. Antibiotic disks (5 mm in diameter) were placed on top of the inoculated agar, ensuring the side containing the antimicrobial agent was facing down to ensure direct contact with the bacteria. The plates were then incubated at 37 °C for 48 h. The inhibition zones around the antibiotic disks were measured every 12 h, following the Kirby–Bauer test protocol [33].

### 2.4. Histopathology

Tissue samples were processed following standard histological procedures [34,35]. Briefly, the tissues underwent a graded dehydration process using ethanol. They were cleared with xylene to eliminate the alcohol and then embedded in paraffin wax to maintain structural integrity. Thin sections (4 μm) of the paraffin-embedded tissue were cut and stained with hematoxylin and eosin (H&E) for general morphological evaluation. The stained sections were subsequently mounted on glass slides under a coverslip for microscopic analysis.

### 2.5. Biomarker of Oxidative Damage

Double-immunofluorescence staining for 4-hydroxynonenal (4-HNE) and 8-hydroxydeoxyguanosine (8-oxodG) was utilized to evaluate the expression of reactive oxygen species (ROS) and to assess DNA damage. Paraffin-embedded tissue sections from lung, liver, spleen, kidney, heart, and brain were processed following the staining protocol described by Jantrakajorn et al. (2024) [36]. An antigen retrieval was performed using a citrate buffer (pH 6.0) in a high-pressure cooker for 10 min. To block non-specific binding, the sections were incubated with 1% normal horse serum in PBS for 45 min at room temperature. After blocking, the tissue sections were incubated overnight at 4 °C with a mixture of two primary antibodies. The first primary antibody, monoclonal mouse anti-4-hydroxynonenal (ab48506, Abcam, Cambridge, UK), was diluted 1:50, while the second, polyclonal goat anti-8-hydroxydeoxyguanosine (AB5830, Sigma-Aldrich, St. Louis, MO, USA), was used at a dilution of 1:200. Following incubation with the primary antibodies, the sections were washed with PBS and incubated with a mixture of secondary antibodies raised in different species: DyLight-488 Donkey Anti-mouse IgG (H + L) (Invitrogen, Waltham, MA, USA) and CF™ 555 Rabbit Anti-goat IgG (H + L) (Sigma-Aldrich, St. Louis, MO, USA), both diluted in 1% BSA, for 1 h at room temperature in the dark. The sections were then mounted with ProLong™ Gold Antifade Mountant containing DAPI (Thermo Fisher Scientific, Waltham, MA, USA) for DNA counterstaining. Fluorescence imaging was performed using a fluorescence microscope (ECLIPSE Ni-U, Nikon, Tokyo, Japan).

### 2.6. AI Tools Statement

Artificial intelligence tools (ChatGPT-4o, OpenAI) were used solely to assist in grammatical correction and language refinement. No AI tools were used for data analysis, content generation, or interpretation of results.

## 3. Results

### 3.1. Anamnesis, Clinical Examination, and Treatment

**Figure 1:**
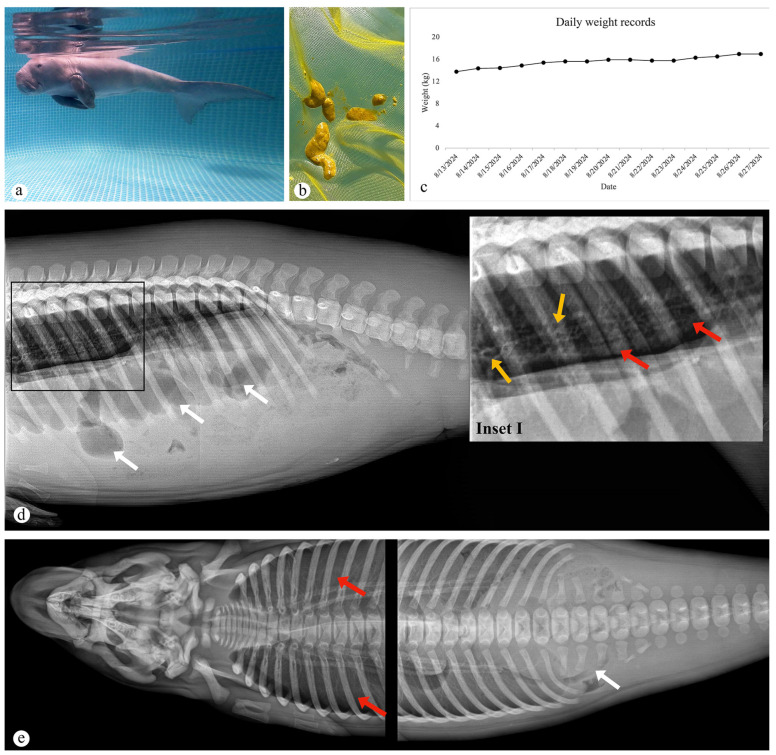
Captive rehabilitation and medical examination of the dugong calf (*Dugong dugon*): (**a**) The dugong was rehabilitated in a fiber circular tank; (**b**) Yellow, soft, unformed feces were present in piles, indicating diarrhea; (**c**) A graph depicting the daily weight of the dugong over a 17-day rehabilitation period; (**d**) Right lateral thoracolumbar radiograph showing an alveolar pattern (inset I, red arrow), bronchial pattern (inset I, yellow arrow) in the lung lobes, and intestinal gas (white arrow); (**e**) Dorsoventral thoracolumbar radiograph showing similar alveolar lung changes (red arrow) and intestinal gas (white arrow).

Following rescue, the dugong calf was transferred to a rehabilitation facility in Trang Province, Thailand. The calf was housed in a fiber circular tank, 1.20 m in depth with a total volume of 28,495 L, filled with natural saltwater (salinity 30 ppt) and a pH around 7.7 (Figure 1a). The water utilized for the care of the dugong calf was stored in a holding tank for 24 h prior to being transferred into the enclosures. There were two enclosures, which are used alternately on a daily basis. Water changes were performed at 100% capacity every day to maintain optimal conditions. The water was filtered continuously using sand filters, and warm water was added to maintain temperature at around 30 °C. The feeding regime initially consisted of a milk replacer formula for the first week. To mimic natural behavior where dugong mothers introduce seagrass to their calves, the veterinarian offered one or two leaves of lettuce prior to each tube feeding, as a form of behavioral enrichment and feeding stimulation. In the initial stage of rehabilitation, the dugong calf was primarily fed a milk formula consisting of 100 g of Milk Matrix 30/52, 100 g of puppy milk, 300 mL of coconut milk, and 35 mL of canola oil, all of which provided an energy intake of 50–80 kcal/kg per day. The formula was divided into four meals, given at 8 a.m., 12 p.m., 5 p.m., and 11 p.m., with a maximum of 300 mL of formula per feeding. In addition, each feeding was supplemented with B-complex vitamins, taurine, lactobacillus, and simethicone to support optimal health and development. After six days of formula feeding, the dugong calf exhibited symptoms of mild diarrhea, characterized by softer stools compared to those of healthy dugongs, as described by Ooi et al. [37], probably attributable to the lactose content in the puppy milk (Figure 1b). Consequently, experts recommended modifying the nutritional formula by eliminating the puppy milk and substituting it with 75 to 140 g of Milk Matrix 30/52 (adjusted according to the calf’s body weight), 450 mL of coconut milk, and 35 mL of canola oil, administered in three divided feedings per day. The final feeding was replaced with 30 g of Oxbow (Critical Care—Herbivore, Oxbow Enterprises, Inc., Omaha, NE, USA) to enhance the calf’s nutritional profile. This formulation adjustment was designed to minimize lactose consumption and optimize digestive function, supporting the calf’s overall health and recovery during the rehabilitation process. Following this adjustment, the calf’s stool returned to normal, with the feces appearing as soft, light yellowish pellets. The dugong’s weight and overall condition gradually improved. However, on the morning of 27 August 2024, the dugong began to show signs of lethargy, floating still, and short breathing, and its breathing rate gradual increased beyond its normal respiratory range. After that, the calf had convulsions and pale mucous membrane, and the heart rate dropped before death on 28 August 2024. The total rehabilitated period was 17 days, and the daily weight record is shown in Figure 1c.

Upon admission, the dugong calf presented with severe dehydration and marked corneal opacity in the left eye. Within 24 h, initial stabilization efforts were made, including the administration of fluids and electrolytes to address the dehydration. Diazepam was also given for muscle relaxation, which resulted in an improvement in the animal’s respiratory rate. Ophthalmic examination revealed significant corneal opacification and ulcerative keratitis with keratomalacia in the left eye, while the right eye appeared normal. The dugong was treated with the antibiotic, levofloxacin 0.5% (Cravit^®^ ophthalmic solution Cravit, Santen Pharmaceutical Co., Osaka, Japan), four times daily. The dimension of the corneal opacity appeared reduced, and the calf showed a positive response to treatment. Blood tests revealed hypoproteinemia due to dehydration, elevated blood urea nitrogen (BUN) levels, and mild leukocytosis. Consequently, topical eye treatment was continued, and nutrition was managed via force-feeding through a stomach tube. The animal’s condition remained stable, with normal heart and respiratory rates. However, on 27 August 2024, the dugong’s condition deteriorated after the water temperature dropped to 27 °C due to rainfall. Initially active, the calf began showing signs of distress around 4:30 a.m., with an increased respiratory rate and body tremors by 5 a.m. By 10:30 a.m., the respiratory rate had risen to over two breaths per minute, and additional signs of weakness were noted throughout the day, indicating a progressive decline in its health.

The right lateral thoracolumbar radiograph of the dugong revealed an alveolar and bronchial pattern in the lung lobes (Figure 1d, inset I), while the dorsoventral thoracolumbar radiograph showed similar alveolar changes involving both lung lobes (Figure 1e). In the abdomen, radiographic findings demonstrated radiopaque areas with gas-filled intestines floating within the peritoneal cavity (Figure 1d,e), suggestive of hydroperitoneum. The dugong showed increased pain and stiffness. A blood glucose test showed significantly decreased levels. Fluids were administered by an oral route and intravenous administration was also attempted. Pain medication and antibiotics were given. Immediate medication was administered, including diazepam (0.1 mg/kg, IM, SID), butorphanol (0.1 mg/kg, IM, SID), and enrofloxacin (5 mg/kg, IM, SID) up until death. Biochemical analyses showed severe hypoalbuminemia (1 g/L; reference ranges 17–47 g/L) and an increased BUN level (16 mg/dL; reference ranges 9–14 mg/dL). The fecal smear showed no evidence of parasitic infestation. Serum biochemistry reference intervals for live wild dugongs were based on the study by Lanyon et al. (2015) [38].

### 3.2. Necropsy and Histopathology Investigations

**Figure 2:**
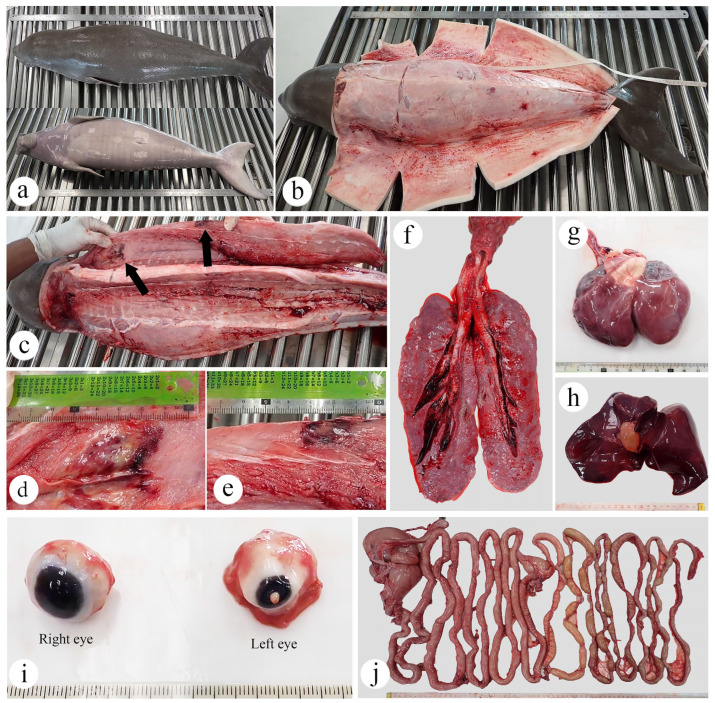
Gross necropsy findings of a dugong calf, *Dugong dugon*: (**a**) Dorsal and ventral views of the body; (**b**) Carcass opening revealing a thick layer of blubber; (**c**) Intramuscular hemorrhage and necrosis; (**d**) Close-up of deep myonecrosis in the anterior region; (**e**) Close-up of deep myonecrosis in the medial region; (**f**) Pulmonary edema and hemorrhage; (**g**) Fluid accumulation on the epicardium; (**h**) Hepatic congestion with gallbladder distention; (**i**) Corneal ulceration with debris material in the left eye; (**j**) Congested intestines.

The postmortem evaluation indicated that the dugong calf’s nutritional status was good (Figure 2a,b). Macroscopic examination of the skin and blubber throughout the body revealed no significant gross lesions. However, the right flipper above the elbow had localized subcutaneous congestion. Additionally, the musculature along the right dorsum had two areas of hemorrhage and necrosis, each approximately measuring 4 × 3 cm (Figure 2c,e). The pericardial sac contained 2.2 mL of serous fluid. Over 80% of the epicardium was contoured and edematous (Figure 2g). The cardiac chambers contained red blood clots. The lungs appeared grayish-pink, firm, mildly edematous, and non-collapsible, with blood oozing from the cut surface (Figure 2f). The trachea was fully occluded by frothy, turbid, dark red fluid and clots, particularly at the distal portion. The abdominal cavity contained 35 mL of serosanguineous fluid. The liver was congested, firm, and mottled dark brown to red (Figure 2h). The gallbladder was markedly distended, containing 7.5 mL of yellowish bile, with limited bile flow; however, no evidence of biliary tract obstruction was observed. The left eye was moderately shrunken and revealed corneal ulceration, characterized by an opened wound with superficial debris material, surrounded by corneal vascularization and granulation tissue (Figure 2i). The spleen had a rounded, bean-shaped appearance, and its cut surface exhibited diffuse reddening. Both kidneys and lymph nodes appeared unremarkable upon examination. Moderate congestion was noted in both the brain and intestines (Figure 2j).

#### 3.2.1. Skin and Muscle

**Figure 3:**
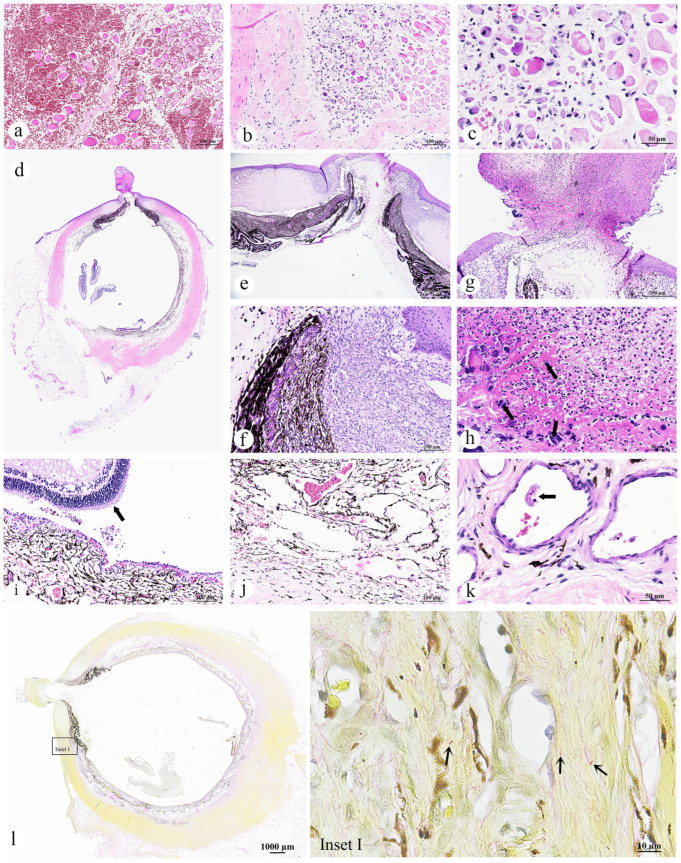
Photomicrographs of the muscular myonecrosis, atrophy, degeneration and hemorrhage, and corneal perforation of a dugong calf (*Dugong dugon*): (**a**) Interstitial hemorrhage of the musculature; (**b**) Muscle fiber degeneration and necrosis; (**c**) Damaged myofibers displayed a range of degenerative alterations, including variations in fiber size, swelling, hypereosinophilia, vacuolation (myofibrillolysis), and necrotic changes; (**d**) Subgross magnification view showing the entire globe with corneal perforation subjacent to eosinophilic debris, along with the presence of anterior synechia; (**e**) Higher magnification of the anterior synechia; (**f**) Adhesion of the iris to the corneal stroma; (**g**) Close-up view of the adhesion site between the corneal debris and the globe; (**h**) Presence of bacterial colonies within the debris material (arrow); (**i**) Retinal detachment (arrow), with hypertrophy of the retinal pigmented epithelial layer; (**j**) The underlying choroid appeared congested, with mild hemorrhage, and was moderately expanded due to edematous fluid; (**k**) Fibrin thrombi in the vascular lumen (arrow); (**l**) Gram staining of the eye tissue showing a positive staining for Gram-negative rod-shaped bacteria (inset I, arrow) at the suspected site of infection. ((**a**–**k**) H&E staining; original magnification: (**d**) Subgross magnification, (**e**) ×4, scale bar = 500 µm; (**a**,**b**,**g**) ×10, scale bar = 250 µm; (**f**,**i**,**j**) ×20, scale bar = 100 µm; (**c**,**h**,**k**) ×40, scale bar = 50 µm; (**l**) gram staining; original magnification: (**l**) Subgross magnification, (**e**) ×4, scale bar = 1000 µm; (**l**) inset I ×100, scale bar = 100 µm).

The skin of the dugong calf exhibited no significant gross lesions. Histopathological examination of the grossly hemorrhaged musculature revealed regionally extensive interstitial hemorrhage (Figure 3a). Muscle fibers varied in size and were rounded and variably shrunken, with some fibers appearing hyalinized, hypereosinophilic, or flocculent, and displaying central, prominent nuclei (myocyte degeneration, atrophy and regeneration). Occasionally, muscle fibers were fragmented with myocyte nuclear loss and prominent contraction bands (myonecrosis). Mild activation of satellite cells was observed. Infiltration of macrophages was evident in the degenerating and necrotic areas, with these cells containing fragments of broken muscle fibers (Figure 3b,c). Additionally, capillaries were lined by endothelial cells exhibiting hypertrophied nuclei.

Morphological diagnoses included the following:

- Skeletal muscle: Myositis and myonecrosis, multifocal, chronic-active, with myofiber degeneration and atrophy, myocyte regeneration, and histiocytic infiltration.

#### 3.2.2. Eye

The left eye had eosinophilic necrotic material adhered to the outer surface of the ocular globe. Subjacent to this material, a corneal perforation was noted (Figure 3d). Surrounding the perforated site, the corneal stroma was circumferentially infiltrated by dense plump fibroblasts admixed proliferation of small caliber blood vessels, indicative of granulation tissue formation, surrounded by edematous collagenous stroma, infiltration of inflammatory cells, along with pale eosinophilic material, suspected to be fibrin. Inflammatory cells were principally lymphocytes, macrophages, and neutrophils. These components appeared to pull the iridal membranes cranially to appose to the cornea and caused the development of anterior synechia (Figure 3e,f). The rest of the corneal stroma had evidence of chronic pigmentary keratitis with epithelial hyperplasia and neovascularization, depicting by pigmentary incontinence, as well as formation of blood vessels scattered throughout. The exophytic eosinophilic material (Figure 3g,h) was composed of cellular debris, fibrin and bacterial colonies, consisting of cocci and bacilli (Figure 3h, arrow), suggesting a secondary infection. Such lesions are commonly associated with trauma or infection. The retina of left eye was detached, exhibiting hypertrophy (tombstoning) of the underlying retinal pigmented epithelium (Figure 3i, arrow). The underlying choroid appeared congested, with mild hemorrhage, and was moderately expanded due to edematous fluid (Figure 3j). Fibrin thrombi were present within the vascular lumen of this layer (Figure 3k). In contrast, the right eye showed no significant pathological findings. Gram staining of the eye revealed colonies of discrete Gram-negative rod-shaped bacteria at the inner choroid layer (Figure 3l, inset I).

Morphological diagnoses included the following:

- Left eye: Kerato-uveitis, chronic-active, suppurative and necrotizing, with corneal perforation, anterior synechia, retinal detachment, fibrin thrombi, and bacterial colonization (Gram-negative bacilli);
- Right eye: No significant lesions.

#### 3.2.3. Gastrointestinal Organ and the Accessory Digestive Structures

**Figure 4:**
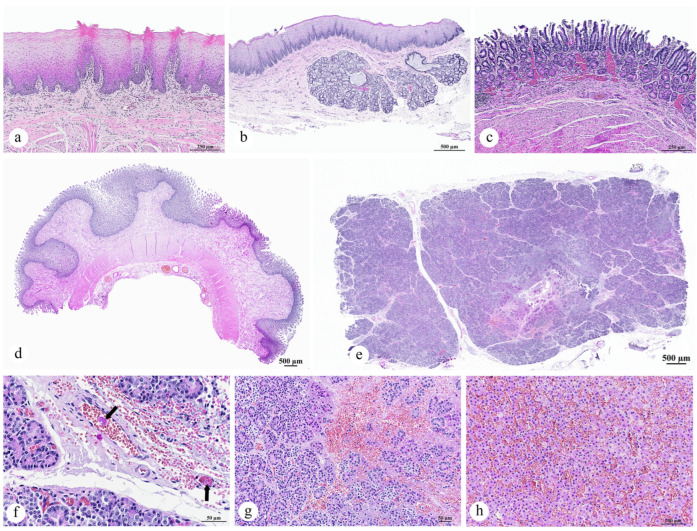
Photomicrographs of the gastrointestinal organs and accessory digestive structures of a dugong calf (*Dugong dugon*): (**a**) No significant changes observed in the architectural structure of the tongue; (**b**) No significant changes observed in the architectural structure of the esophagus; (**c**) Gastric congestion; (**d**) Intestinal congestion; (**e**) Moderate peracute pancreatic necrosis; (**f**) Presence of fibrin thrombi in the pancreatic interlobular capillaries (arrow); (**g**) Area of hemorrhage and necrosis within the pancreas; (**h**) Severe hepatic congestion. ((**a**–**h**) H&E staining; original magnification: (**b**,**d**,**e**) subgross magnification; (**a**,**c**) ×10, scale bar = 250 µm; (**h**) ×20, scale bar = 100 µm; (**f**,**g**) ×40, scale bar = 50 µm).

Histopathological evaluation of the tongue, esophagus, stomach, intestine, liver, gallbladder, and pancreas revealed the following findings. No significant changes were observed in the tongue (Figure 4a). The esophagus had a mild thickening of keratinous layers with retained nuclei (parakeratotic hyperkeratosis) (Figure 4b). Examination of the stomach and intestinal tissues showed no remarkable mucosal alterations, although moderate congestion of the vasculature was evident (Figure 4c,d). The pancreas exhibited lysis of the pancreatic parenchyma, though this did not extend into the peripancreatic adipose tissue. Areas of lytic necrosis were multifocal to coalescing, characterized by the loss of cellular architecture and replacement with eosinophilic cellular debris, karyorrhectic material, fibrin, hemorrhage, and edema (Figure 4e). Notably, no rimmed necrotic neutrophils or macrophage reactions were observed, suggesting a peracute necrosis process. Within the adjacent pancreatic parenchyma, acinar cells were either swollen with pale, vacuolated eosinophilic cytoplasm (indicating degeneration) or shrunken with hypereosinophilic cytoplasm, pyknotic nuclei, and the loss of zymogen granules (indicating single-cell death). Additionally, prominent changes were noted in the pancreatic vasculature, including interlobular capillaries, pancreatic arterioles, and small-caliber capillary vessels. The interlobular capillaries displayed discrete to extensive amorphous or meshwork-like deposits of finely granular, eosinophilic material (peracute fibrin thrombi), which were closely associated with lytic necrosis (Figure 4f, arrow). Within the necrotic foci, blood vessel vascular tunics were transmurally expanded by scant necrotic cellular debris, fibrin, hemorrhage, and edema (Figure 4g). In the liver, severe congestion was observed in scattered panlobular regions, accompanied by hepatocellular degeneration (Figure 4h).

Morphological diagnoses including the following:

- Pancreas: Pancreatic necrosis, lytic, multifocal to coalescing, peracute, with fibrin thrombi and vascular necrosis;
- Liver: Congestion, multifocal to coalescing, moderate to severe, with hepatocellular degeneration;
- Esophagus: Parakeratotic hyperkeratosis, mild;
- Stomach and intestine: Congestion, mild;
- Tongue: No significant lesions.

#### 3.2.4. Lymph Node, Thymus, and Spleen

**Figure 5:**
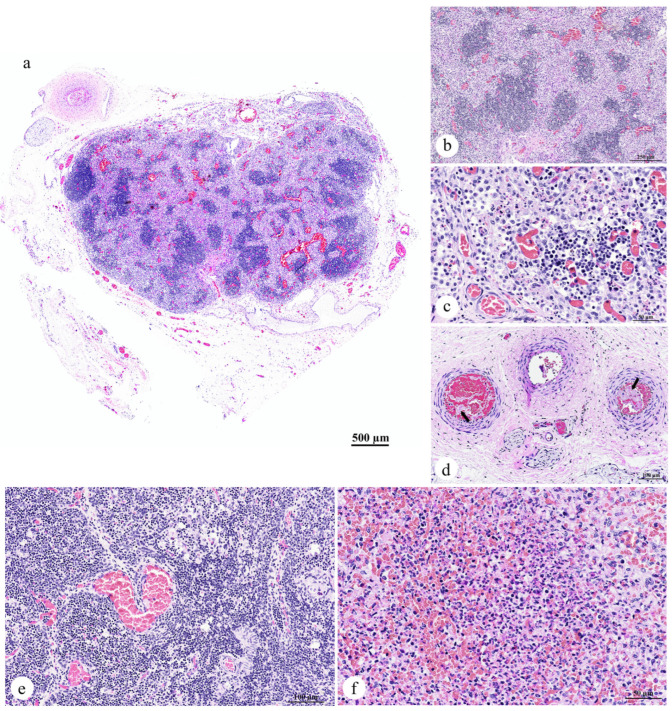
Photomicrographs of the lymphatic organs of a dugong calf (*Dugong dugon*): (**a**) Subgross magnification view showing expansion of the lymph node; (**b**) Higher magnification of the paracortical hyperplasia; (**c**) Lymph node congestion and infiltration of neutrophils in the paracortex; (**d**) Presence of fibrin thrombi in the lymphatic arterioles (arrow); (**e**) No significant changes observed in the architectural structure of the thymus; (**f**) Severe acute multifocal neutrophilic and necrotizing splenitis. ((**a**–**f**) H&E staining; original magnification: (**a**) subgross magnification; (**b**) ×10, scale bar = 250 µm; (**d**,**e**) ×20, scale bar = 100 µm; (**c**,**f**) ×40, scale bar = 50 µm).

The axillary lymph node exhibited marked enlargement, characterized by an increased accumulation of macrophages within the paracortex of the lymph node tissue, leading to paracortical hyperplasia (Figure 5a,b). Aggregates of neutrophils were often observed infiltrating the paracortex (Figure 5c). Additionally, prominent vascular congestion was observed throughout the lymph node (Figure 5c). The lymphatic vessels contained amorphous fibrin thrombi (Figure 5d, arrow). No notable alterations were detected in the thymus (Figure 5e). Examination of the spleen revealed a reduction in the size of the white pulp follicles, which were widely spaced by hypercellular red pulp. This red pulp was composed of erythrocytes interspersed with abundant homogeneous eosinophilic material, likely representing lysed erythrocytes. Furthermore, numerous neutrophils and scattered cellular debris were noted (Figure 5f).

Morphological diagnoses included the following:

- Lymph node: Lymphadenitis, neutrophilic and histiocytic, acute, multifocal to coalescing, with paracortical hyperplasia, vascular congestion, and fibrin thrombi;
- Spleen: Splenitis, neutrophilic and necrotizing, acute, multifocal, severe, with red pulp hypercellularity and white pulp depletion;
- Thymus: No significant lesions.

#### 3.2.5. Cardiorespiratory System

**Figure 6:**
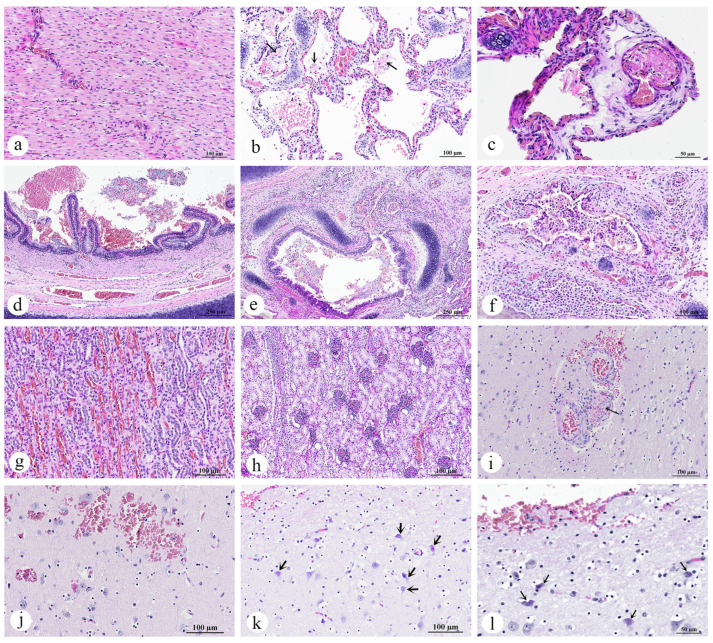
Photomicrographs of the heart, lung, kidney, and brain of a dugong calf (*Dugong dugon*): (**a**) The heart with no significant architectural changes; (**b**) Desquamated fetal skin cells (arrow) observed within an alveolus, alongside hemorrhage and epithelial detachment; (**c**) Fibrin thrombi in the alveolar capillaries (arrow); Excessive mucus mixed with red blood cells and epithelial debris in tracheal lumen (**d**), bronchial lumen (**e**); (**f**) Inflammation of the bronchus and adjacent pulmonary parenchyma; (**g**) Congestion noted in the renal medulla; (**h**) Congestion observed in the renal cortex and glomeruli; (**i**) Fibrin thrombi in a brain venule (arrow); (**j**) Hemorrhage in the neuroparenchyma; (**k**) Neuronal degeneration and necrosis (arrow); (**l**) Higher magnification of neuronal degeneration and necrosis (arrow). ((**a**–**l**) H&E staining; original magnification: (**d**,**e**) ×10, scale bar = 250 µm; (**a**,**b**,**f**–**k**) ×20, scale bar = 100 µm; (**c**,**l**) ×40, scale bar = 50 µm).

Histopathological examination of the dugong calf revealed no significant lesions in the heart (Figure 6a). However, the trachea and lung sections exhibited notable alterations. In the lung, approximately 75% of the tissue exhibited congestion, with minimal hemorrhage. These regions contained dense accumulations of erythrocytes, along with aggregates of eosinophilic proteinaceous edema fluid, plump vacuolated macrophages, and epithelial detachment. Flattened “squames” or desquamated fetal skin cells, typically found in the amniotic fluid, were observed within an alveolus, alongside hemorrhage and epithelial detachment (Figure 6b). Additionally, marked changes were observed in the pulmonary vasculature, including the arterioles and alveolar capillaries, which showed discrete to extensive deposits of amorphous eosinophilic material, suggestive of peracute fibrin thrombi (Figure 6c). In the trachea, there was excessive mucus accumulation, mixed with epithelial debris and extravasated red blood cells (Figure 6d). In the bronchial lumens, a moderate accumulation of macrophages, lymphocytes, and neutrophils, along with mixed mucus, was observed (Figure 6e). The bronchial mucosa showed mild infiltration by lymphocytes, histiocytes and neutrophils. Inflammatory cells had transmigrated through the epithelium, extending into the submucosa and adjacent pulmonary parenchyma, and variably filling the bronchiolar and parabronchial areas (Figure 6f).

Morphological diagnoses included the following:

- Lung: Pulmonary congestion and edema, acute, multifocal to coalescing, with pulmonary hemorrhage, intra-aveolar squames (aspiration), and fibrin thrombi;
- Trachea: Intraluminal mucus and epithelial debris;
- Bronchi/bronchioles: Bronchopneumonia, lymphohistiocytic and neutrophilic, mild to moderate, with mucosal and submucosal inflammation;
- Heart: Congestion, no significant lesions.

#### 3.2.6. Adrenal Glands, Kidneys, and Urinary Bladder

The adrenal glands, kidneys, and urinary bladder exhibited minimal pathological changes. Notable congestion was observed primarily in the vasculature and glomeruli of the kidneys, affecting both the renal cortex and medulla (Figure 6g,h). However, no fibrin thrombi were detected within the renal vasculature.

Morphological diagnoses included the following:

- Kidneys: Congestion, multifocal, mild to moderate;
- Urinary bladder: No significant lesions;
- Adrenal glands: No significant lesions.

#### 3.2.7. Brain

Histopathological examination of the cerebrum, brainstem, and cerebellum revealed that some vessels, primarily veins and venules, contained well-organized thrombi composed of neutrophils and red blood cells (Figure 6i). The endothelium of the affected vessels was disrupted, and expanded by fibrinoid material, as well as areas of frank hemorrhage extending beyond the vessel walls. The surrounding neuroparenchyma exhibited microhemorrhage (Figure 6j). There was no evidence of inflammation in the affected vessels. Neurons in the affected regions exhibited cellular alterations and cell death, characterized by degeneration, increased eosinophilia, and karyolysis (Figure 6k,l).

Morphological diagnoses included the following:

- Cerebrum, brainstem, and cerebellum: Thromboembolic vasculopathy with fibrinoid necrosis and microhemorrhages, multifocal, with neuronal degeneration and necrosis.

The histopathological findings are summarized in Table 1 to facilitate clearer visualization and comparison of lesion types across different tissues.

### 3.3. Bacteriological Analysis and Antibiotic Susceptibility Testing

Bacteriological analysis was conducted on samples obtained from major organs, including the lung, liver, spleen, kidney, heart, and brain. The results revealed the growth of a single bacterial colony on the agar plates. Subsequent sub-culturing and Gram staining identified the bacterium as a Gram-negative rod, which was further characterized as *Achromobacter xylosoxidans* through species identification. Antibiotic susceptibility testing indicated that the isolate was resistant to amikacin, cefazolin, ceftriaxone, and oxytetracycline. It exhibited intermediate resistance to enrofloxacin and marbofloxacin, while being susceptible to amoxicillin–clavulanic acid, doxycycline, imipenem, and sulfa-trimethoprim (Table 2). The resistance profile of *Achromobacter xylosoxidans* in this case indicates limited efficacy of commonly used antibiotics such as aminoglycosides, cephalosporins, and oxytetracycline. Intermediate resistance to fluoroquinolones further complicates treatment choices. While susceptibility to drugs like amoxicillin–clavulanic acid and imipenem offers potential options, this highlights the need for culture-based antibiotic selection in dugong rehabilitation to improve therapeutic success and minimize resistance development.

### 3.4. Immunofluorescence Analysis of Oxidative Damage

**Figure 7:**
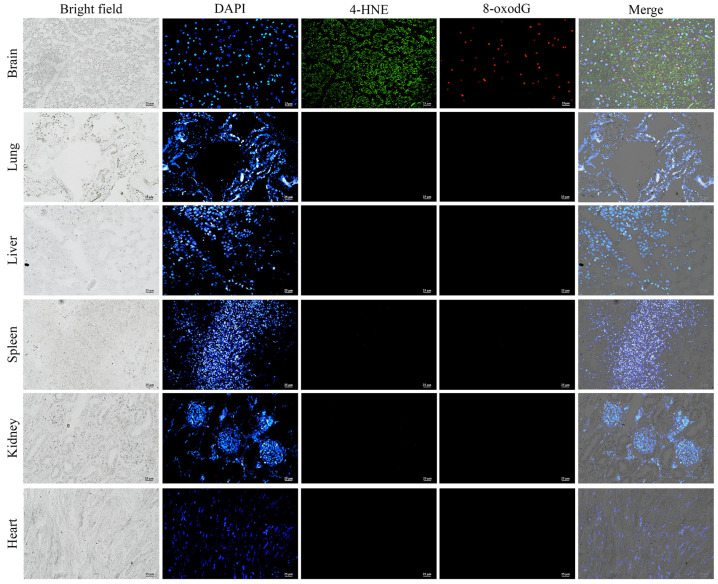
Expression of reactive oxygen species (4-HNE) and damaged DNA (8-oxodG) in different organs of the *Achromobacter xylosoxidans*-infected dugong calf. Panels from left to right show bright field, DAPI-stained nuclei, 4-HNE labeled with DyLight-488 (Invitrogen, Waltham, MA, USA (green), 8-oxodG labeled with CF™ 555 (Sigma-Aldrich, St. Louis, MO, USA) (red), and the merged image of the four panels. Co-localization of 4-HNE (green), 8-oxodG (red), and DAPI (blue) was observed exclusively in the brain tissue of the affected dugong calf. The purple spots represent the co-localization of damaged nuclei associated with DNA damage. (Immunofluorescence: DyLight-488 for 4-HNE (green), CF™ 555 for 8-oxodG (red), DAPI counterstain for nuclei (blue); original magnification, ×40; scale bar represents 50 μm).

Immunofluorescence staining was performed using 4-hydroxynonenal (4-HNE) and 8-hydroxydeoxyguanosine (8-oxodG) markers to investigate the impact of peracute DIC-induced oxidative stress and DNA damage on cellular structures in major organs, including the lung, liver, spleen, kidney, heart, and brain. Based on visual assessment, cells positive for 4-HNE exhibited a cytoplasmic staining pattern, whereas 8-oxodG-positive cells displayed nuclear staining. In the brain, 4-HNE expression was initially detected in neuronal cells, with increased expression observed in regions displaying heightened neuronal degeneration. A significant upregulation of 8-oxodG expression was also identified in the brain. Notably, areas with intense 4-HNE expression also exhibited prominent co-expression of 8-oxodG, indicating concurrent oxidative damage and DNA damage in these regions. In contrast, no expression of either 4-HNE or 8-oxodG was detected in the lung, liver, spleen, kidney, or heart tissues. These findings suggest that, in the context of per-acute DIC in the dugong calf, the brain is the primary organ affected by oxidative stress and DNA damage (Figure 7). The co-localization of 4-HNE and 8-oxodG in neuronal regions suggests that oxidative stress and DNA damage may play a crucial role in initiating or exacerbating neuronal death, contributing to the observed mortality. While oxidative damage has the potential to drive neuroinflammation, such a response typically requires sufficient time for inflammatory cells to infiltrate and respond to free radical-induced injury. Therefore, in this peracute case, the lack of inflammatory cell infiltration likely reflects the rapid progression of the disease, which limited the development of a typical neuroinflammatory response.

## 4. Discussion

This study presents the first documented case of acute systemic infection and DIC in a dugong calf associated with *Achromobacter xylosoxidans*. This bacterium is an opportunistic Gram-negative organism commonly found in aquatic environments. [39]. It is a well-recognized pathogen in humans, known to cause sepsis and septicemia, particularly in neonates and immunocompromised individuals [40,41,42]. Although reports of *A. xylosoxidans* infection in animals are limited, it has been implicated in cases of endocarditis and joint disease in dogs [43,44]. To the best of our knowledge, this is the first report of *A. xylosoxidans* infection in a dugong and the first to demonstrate its potential to induce DIC in marine mammals. This finding is particularly noteworthy in the context of systemic bacterial infections, which are relatively common among sirenians, particularly in manatees. Several bacterial pathogens have been identified in the Florida manatee (*Trichechus manatus latirostris*), including *Salmonella enterica*, which has been associated with lesions in the renal, respiratory, lymphatic, and skeletal systems [45]. *Clostridium* spp. have also been implicated in cases of septicemia and endotoxemia [46], while *Mycobacterium marinum* and *M. kansasii* have been reported to cause disseminated tuberculosis [47]. However, none of these previous reports have involved *A. xylosoxidans*, highlighting the novelty and significance of the present case. In addition to identifying the pathogen, this study also characterized its antimicrobial resistance profile, which has important implications for postmortem diagnosis, epidemiological surveillance, and understanding the potential risks posed by opportunistic aquatic pathogens. Antibiotic susceptibility testing demonstrated a multidrug-resistant profile, with the isolate exhibiting resistance to several commonly used antibiotics, including amikacin, cefazolin, ceftriaxone, and oxytetracycline. This resistance pattern aligns with previously reported findings [48,49], which attribute antimicrobial resistance in *A. xylosoxidans* to both intrinsic and acquired mechanisms, such as the production of β-lactamases, overexpression of efflux pumps, and biofilm formation, all of which contribute to significant therapeutic challenges [50,51]. In this case, the pathogen’s dissemination, combined with the calf’s immature immune system, is likely to have accelerated the onset of DIC and multi-organ failure. Impairment of immune system function increases susceptibility to opportunistic infections, particularly in neonates or immunocompromised individuals [52]. As reported in similar studies on cetaceans, such immunosuppression may result from synergistic factors including stress, exposure to heavy metals and environmental pollutants, or concurrent infections [53,54,55]. These findings underscore growing concerns regarding environmental influences on host immunity, the emergence of antimicrobial resistance in aquatic pathogens, and the need for targeted therapeutic strategies in vulnerable marine mammals.

Furthermore, this case emphasizes the importance of reviewing and enhancing rehabilitation protocols to minimize the risk of opportunistic bacterial contamination. Potential sources of *A. xylosoxidans* in the rehabilitation environment may include contaminated water supplies, feeding equipment, or even human handling [56,57]. Given the bacterium’s ubiquitous presence in aquatic settings and its ability to form and persist in biofilms [58], special attention should be given to hygiene practices, sterilization procedures, and water quality management within rehabilitation facilities. To better understand and control such infections, further systematic environmental screening for bacterial contaminants should be conducted, particularly on water systems and feeding apparatus, to help identify the origin of infection and guide appropriate preventive strategies.

DIC is characterized by systemic activation of coagulation, impaired anticoagulant pathways, and suppression of fibrinolysis, leading to widespread microvascular thrombosis followed by consumptive coagulopathy and multiple organ dysfunction syndrome [59]. In our study, extensive histopathological lesions were observed, particularly, fibrin thrombi in the pulmonary, pancreatic, and cerebral vessels, along with hemorrhage and necrosis in multiple organs, leading to multiorgan failure and death. These findings are hallmark features of DIC [60]. Additionally, severe ocular lesions, including corneal perforation with Gram-negative bacterial colonization and retinal detachment, suggest a localized initial site of infection that may have served as a portal for systemic bacterial dissemination. Previous studies have indicated that microbial colonization following ocular trauma or via hematogenous spread can facilitate the onset of systemic infections [61]. Moreover, hepatobiliary sepsis has been reported in human cases of ocular infections, supporting a possible association between ocular pathology and systemic bacterial invasion [62]. Thus, the ocular lesions observed in this dugong calf may represent portal of entry, an early clinical indicator of an underlying, progressing systemic infection. Despite supportive care during rehabilitation, the calf’s immune immaturity, indicated by severe leukocytosis and hypoalbuminemia. This pattern is associated with the acquisition and severity of infectious diseases, and intact innate and adaptive immune responses [63]. Like in other young animals, this neonate possessed underdeveloped immune mechanisms, rendering it susceptible to opportunistic pathogens under environmental or nutritional stress [64,65]. These immunological limitations probably contributed to the progression of infection and the eventual onset of fatal DIC in this case.

The pathogenesis observed in this case is consistent with microvascular thrombosis, which probably resulted in tissue hypoxia, subsequently triggering inflammatory responses and activation of coagulation cascade mechanisms previously described in the context of DIC [59]. A key component of this pathological process appears to be hypoxia-induced oxidative stress, which damages the vascular endothelium and leads to elevated intracellular levels of reactive oxygen species (ROS) [66,67]. Notably, dysfunction of the mitochondrial electron transport chain under hypoxic conditions is considered a major source of excessive ROS production [61]. These elevated ROS levels can inflict significant damage to cellular components, including lipids, proteins, and DNA [68]. In the present study, immunofluorescence staining indicated this pathophysiological mechanism by revealing marked oxidative and DNA damage, particularly within the brain tissue. This localization suggests that the brain was a primary target of systemic dysfunction and is likely to have contributed substantially to the mortality observed in this case.

## 5. Conclusions

This case represents the first documented occurrence of *Achromobacter xylosoxidans*–associated septicemia leading to DIC in a dugong (*Dugong dugon*) calf. The findings underscore the increased susceptibility of juvenile dugongs, whose immunological immaturity predisposes them to opportunistic environmental pathogens. The rapid progression from the onset of clinical signs to fatal systemic infection emphasizes the necessity for prompt diagnostic investigation and immediate therapeutic intervention. Furthermore, this report highlights the critical importance of rigorous biosecurity and infection control measures within rehabilitation facilities to mitigate the risk of pathogen exposure. It also supports the implementation of vigilant monitoring, early diagnostic testing, and prophylactic strategies. These observations contribute valuable insights to the limited literature on dugong pathology and carry important implications for improving clinical management and conservation efforts for stranded marine mammals.

## Figures and Tables

**Table 1:** Comparative summary of lesion types observed in different organs of the dugong calf.

Tissue/Organ	Congestion	Hemorrhage	Necrosis	Cellular Infiltration	Fibrin Thrombi in Capillary
**Musculoskeletal system**					
Skeletal muscle	✓	✓	✓	✓	
**Nervous and sensory system**					
Brain	✓	✓	✓		✓
Eye	✓	✓		✓	
**Digestive system**					
Tongue	✓				
Esophagus	✓				
Stomach	✓				
Intestine	✓				
Pancreas	✓	✓	✓	✓	✓
Liver	✓				
**Lymphatic system**					
Lymph node	✓			✓	✓
Spleen	✓			✓	✓
Thymus	✓				
**Urinary system**					
Kidney	✓				
Urinary bladder	✓				
**Endocrine system**					
Adrenal gland	✓				
**Cardiorespiratory system**					
Heart	✓				
Trachea	✓	✓		✓	✓
Bronchi/bronchioles	✓	✓		✓	✓
Alveolar	✓	✓		✓	✓

**Table 2:** Bacteriological analysis using VITEK-2 Compact system and antibiotic susceptibility testing the Kirby–Bauer disk diffusion method.

Antibiotic	Concentration (µg) of the Antibiotic in Disc	Zone of Inhibition (mm)		
		Resistant	Intermediate	Sensitive
Amoxicillin–clavulanic acid	20/10	-	-	23
Amikacin	30	12	-	-
Cefazolin	30	13	-	-
Ceftriaxone	30	12	-	-
Doxycycline	30	-	-	20
Enrofloxacin	5	-	19	-
Imipenem	10	-	-	27
Marbofloxacin	5	-	17	-
Oxytetracycline	30	13	-	-
Sulfa-trimethoprim	1.25/23.7	-	-	26

## Data Availability

The data presented in this study are available on request from the corresponding author (W.S.).

## Abbreviations

The following abbreviations are used in this manuscript:

4-HNE	4-hydroxynonenal
8-oxodG	8-hydroxy-2′-deoxyguanosine
DIC	Disseminated intravascular coagulation
DNA	Deoxyribonucleic acid
IM	Intramuscular
IUCN	International Union for Conservation of Nature
ROS	Reactive oxygen species
SID	Semel in die, which is Latin for “once a day” or “once daily”
°C	Degrees Celsius
